# Determinants of non-adherence to adjuvant endocrine treatment in women with breast cancer: the role of comorbidity

**DOI:** 10.1007/s10549-018-4890-z

**Published:** 2018-07-21

**Authors:** W. Wulaningsih, H. Garmo, J. Ahlgren, L. Holmberg, Y. Folkvaljon, A. Wigertz, M. Van Hemelrijck, M. Lambe

**Affiliations:** 1 Translational Oncology and Urology Research (TOUR), School of Cancer and Pharmaceutical Sciences, King’s College London, Guy’s Hospital, 3rd Floor, Bermondsey Wing, London, SE1 9RT UK; 2 MRC Unit for Lifelong Health and Ageing at University College London, London, UK; 3 Regional Cancer Centre Uppsala-Örebro, Uppsala, Sweden; 4 Faculty of Medicine, University of Örebro, Örebro, Sweden; 5 Department of Medical Epidemiology and Biostatistics, Karolinska Institutet, Stockholm, Sweden

**Keywords:** Breast cancer, Endocrine treatment, Adherence, Tamoxifen, Comorbidity

## Abstract

**Purpose:**

To examine factors associated with non-adherence during 5 years of endocrine treatment, including the possible influence of comorbidity burden and specific medical conditions.

**Methods:**

From all women diagnosed with stage I–III, ER-positive breast cancer in Stockholm-Gotland, Uppsala–Örebro and Northern Sweden between 2006 and 2009, we included 4645 women who had at least one dispensation of tamoxifen or aromatase inhibitors (AIs) and 5 years of follow-up without distant recurrence. A medical possession ratio of < 80% was used to define non-adherence. Logistic regression was used to estimate odds ratios (ORs) and 95% confidence intervals (CIs) of non-adherence.

**Results:**

During follow-up, 977 (21%) women became non-adherents. Non-adherence was associated with greater comorbidity burden assessed by Charlson comorbidity index (CCI) during follow-up (OR 1.43; 95% CI 1.08–1.88 for ≥ 2 additional scores compared to 0), pre-diagnostic HRT use (OR 1.99; 1.58–2.49), not married (OR 1.42; 1.23–1.64), high educational level (OR 1.25; 1.02–1.53 compared to lowest level), and use of symptom-relieving drugs. HER-2 positivity (OR 0.61; 0.45–0.81) and adjuvant chemotherapy (OR 0.42; 0.35–0.52) were associated with lower odds of non-adherence. Similar patterns were observed for the presence of lymph node metastasis, higher tumour grade, and use of AIs compared to tamoxifen. Myocardial infarction and chronic pulmonary disease was suggested as leading conditions associated with non-adherence in women with increasing CCI.

**Conclusion:**

We identified subgroups of women with breast cancer at increased risk of non-adherence. Our findings related to comorbidity suggest the importance of focusing on the presence of specific co-existing conditions when monitoring adherence.

## Background

Adjuvant endocrine treatment (ET) is part of the standard therapy for oestrogen receptor (ER)-positive breast cancers [1]. Survival benefit following an extended use of ET has been documented,[2] prompting recommendation for 5–10 years use of ET following a breast cancer diagnosis [3]. On the other hand, low adherence to adjuvant ET in breast cancer may result in shorter time to recurrence, higher medical costs and poorer quality of life [4, 5]. Therefore, an improved understanding of factors affecting patient adherence is of importance when developing intervention strategies. A systematic review including 30 studies investigating non-adherence and/or discontinuation of ET in women with hormone receptor-positive breast cancer have suggested several key determinants, including patient age, out-of-pocket costs, changes of therapy, follow-up and treatment side effects [6, 7]. However, these studies differed with regard to assessment methods, length of follow-up and factors under study [6, 8] Furthermore, while findings from earlier studies have linked multimorbidity with lower adherence to ET, [9] results from most studies have been based on comorbidities assessed at baseline and did not investigate specific types of co-existing disease or changes in comorbidity burden over time.

We have previously reported on non-adherence and early discontinuation patterns of use of adjuvant endocrine treatment among women with breast cancer in Sweden [10]. Following an update of the cohort used in that study, we aimed to further investigate determinants of adherence by extending duration of follow-up to 5 years, and including additional potential determinants ranging from patient to health system-related factors based on the World Health Organization (WHO) multidimensional model for drug adherence [11]. In addition, we assessed the possible influence of comorbidities both quantitatively and qualitatively by looking into specific conditions.

## Methods

### Study population

**Fig. 1:**
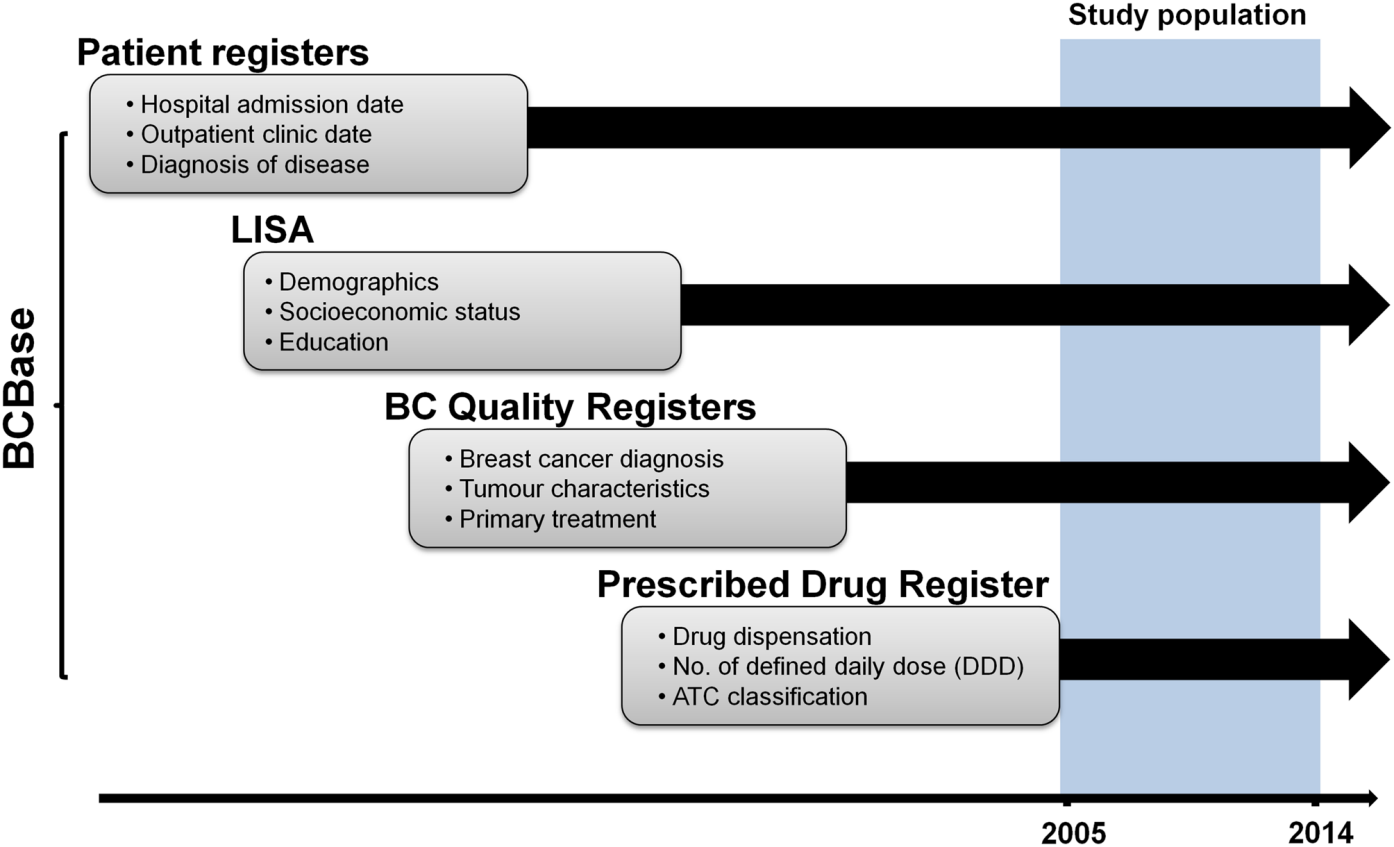
Data sources. Each rectangle illustrates different Swedish registers which were cross-linked using an individually unique 10-digit personal identification number to generate the research database BCBaSe. Shaded area indicates the period of observation between 2005 and 2014 which the present study was based on. Patient Registers: diagnostic codes for inpatient and outpatient care. *BC Quality Registry* Regional Breast Cancer Clinical Quality Registers of the Uppsala/Örebro, Stockholm-Gotland and Northern regions of Sweden. *LISA register* Longitudinal integration database for health insurance and labour market studies. Prescribed Drug Register: details regarding filed drugs

Our study was based on information in the breast cancer research database BCBaSe generated by record linkage between four Swedish population-based registers. Details on period and data covered by each register are shown in Fig. 1. The Regional Breast Cancer Clinical Quality Registers of the Uppsala/Örebro, Stockholm-Gotland and Northern regions of Sweden includes information on women newly diagnosed with breast cancer in these regions with less than 5% missing when validated against the National Swedish Cancer Register to which reporting is mandated. The Swedish Prescribed Drug Register encompasses prescribed medications dispensed in Swedish pharmacies and includes information on dates of dispensation, number of defined daily doses (DDD) and classification of the drugs based on the Anatomic Therapeutic Chemical (ATC) system. Individual-level information on socioeconomic and demographic factors was obtained from the Longitudinal integration database for health insurance and labour market studies (LISA) that integrates existing data from labour market-, educational-, and social-sector registers on all individuals 16 years or older residing in Sweden, and is updated on a yearly basis. Finally, the Swedish Patient Register contains information on hospital admissions and outpatient clinic visits with dates and diagnostic codes. Information from the above registers was linked using a ten-digit personal identifier number assigned to all permanent residents in Sweden.

### Study population and follow-up

**Fig. 2:**
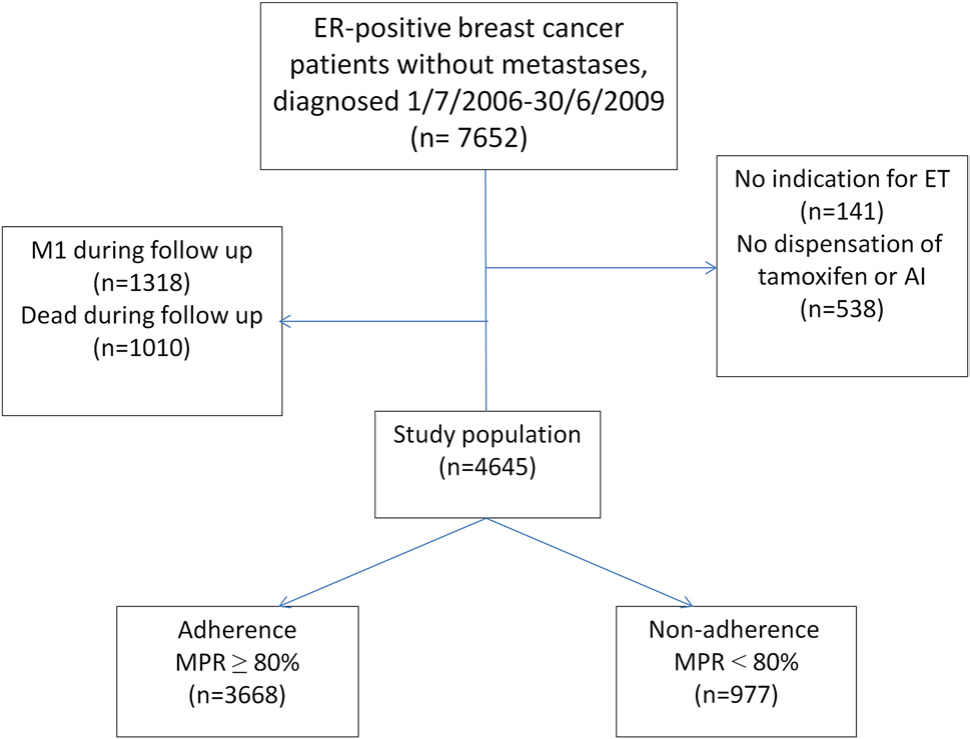
Selection of study participants and non-adherence. Non-adherence was defined as having MPR less than 80% over 5 year of follow-up. *ET* endocrine treatment, *M1* distant metastasis, *MPR* Medical Possession Ratio, calculated by dividing the total defined daily dose (DDD) required during the period of observation by the total number of dispensed DDD

We identified all women residing in the Uppsala/Örebro, Stockholm-Gotland and Northern regions of Sweden with a diagnosis of stage I–III ER-positive invasive breast cancer between July 1, 2006 and June 30, 2009 and at least one dispensation of oral tamoxifen (ATC code: L02BA01) or aromatase inhibitor (AI; anastrozole, exemetastane, or letrozole; ATC code: L02BG) after diagnosis (Fig. 2). To avoid misclassification of adherence status because of early death and metastasis, women who died or had distant metastasis during follow-up were excluded (Fig. 1). We used a run-in period of 6 months to avoid overestimation of adherence to endocrine treatment [12]. This 6-month interval was based on the median duration between breast cancer diagnosis and the initial dispensation of endocrine treatment. Therefore, for all women, follow-up started at the end of run-in period, i.e. 6 months post-diagnosis, and ended 5 years afterwards.

### Adherence to endocrine treatment

Patterns of adherence were assessed based on tamoxifen and AI dispensations as recorded in the Swedish Prescribed Drug Register. To quantify adherence to endocrine treatment, we calculated medical possession ratio (MPR) as the ratio of the number of days for which a patient has medication on hand divided by the total number of days of follow-up, and multiplied by 100%. Non-adherence was defined as having an MPR of < 80%.

### Determinants of adherence

**Fig. 3:**
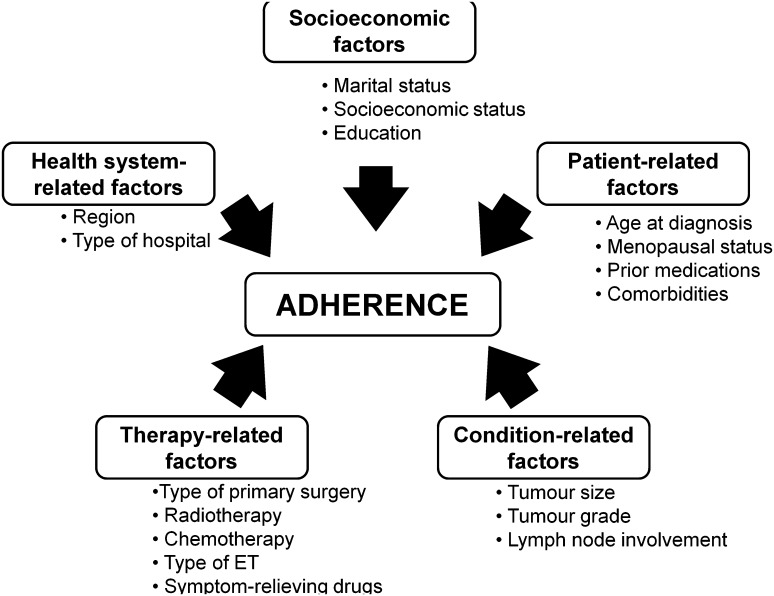
Determinants of adherence to endocrine treatment in breast cancer patients. Each potential determining factor in the study population was plotted against the World Health Organization (WHO) five dimensions of drug adherence

We classified potential determinants of adherence based on the World Health Organization (WHO) multidimensional adherence model (Fig. 3) [11]. Five major factors were considered to influence adherence: socioeconomic, patient-related, condition-related, therapy-related and health system-related factors.

For the purpose of the present study, health system-related factors included region where breast cancers were diagnosed and whether surgery or follow-up was conducted in university hospitals.

Patient-related factors included age at diagnosis, educational level, menopausal status, marital status, comorbidities and prior medications. Educational attainment was categorised into low, medium and high, corresponding to less than high school (≤ 9 years), high school graduate (10–12 years) and higher education (> 12 years), respectively.

Comorbidities were assessed by calculating the Charlson comorbidity index (CCI) [13] based on information on inpatient admissions and outpatient visits 10 years prior to the start of follow-up and data on specific medications (e.g. insulin for diabetes) prescribed during the run-in period. The CCI consists of 17 groups of diseases with a specific weight assigned to each condition, and these weights were then summed to obtain an overall score, resulting in four comorbidity levels (0, 1, 2 and 3+) indicating no comorbidity to severe comorbidity. In addition to CCI assessed at baseline, we also calculated CCI at the end of follow-up and any differences between the two continuous scores (ΔCCI) were estimated.

We collected information on drugs commonly associated with adverse effects of cancer treatments. Included in this category were analgesics (ATC code: N02), hypnotics or sedatives (ATC code: N05C), antidepressants (ATC code: N06A), gastrointestinal (GI) drugs (ATC code: A02–A07) and topical oestrogen for vaginal use (ATC code: G03C). Use of these drugs was calculated during the run-in period to assess baseline use, and during follow-up using information from the Prescribed Drug Register. Baseline use of oral hormone replacement therapy (HRT; ATC code: G03C–G03F) was collected since 1 year prior to breast cancer diagnosis until the end of the run-in period. Drugs used during follow-up may reflect adverse effect of cancer treatment and, therefore, they were classified under treatment-related factors. Other treatment-related factors were type of primary surgery, radiotherapy, chemotherapy and type of endocrine treatment used.

Condition-related factors included tumour size, lymph node involvement, tumour grade based on Elston grading system, progesterone receptor (PR) and human epidermal growth factor receptor 2 (HER2) status.

### Statistical analysis

Univariable logistic regression analysis was performed to estimate odds ratios (OR) and their 95% confidence intervals (CI) of non-adherence to ET for each potential determinant, with women who remained adherent as the reference group. Analyses were repeated following adjustment of age at baseline, and a further analysis was conducted while additionally adjusting for SES. A sensitivity analysis was performed by excluding women without records of any dispensation of ET during the final year of follow-up, representing those who discontinued treatment. To tease out the potential role of specific medical conditions for a given comorbidity group in CCI, Fisher’s exact test was used to assess differences in the proportion of non-adherents by whether women never had, or had the condition at baseline, or were diagnosed with the specific co-existing disease during follow-up.

## Results

A total of 4645 women with ER-positive breast cancers treated with ET were included in our analysis. During the 5-year follow-up, 977 women (21%) became non-adherent, i.e. having MPR < 80%. Table 1 describes study participants by adherence status and potential determinants of non-adherence, and the corresponding estimates from regression analysis. The majority of women were post-menopausal and had completed high school. In univariable analyses, we identified several predictors of non-adherence to ET.

**Table 1:** Determinants of non-adherence to adjuvant endocrine treatment in Swedish women diagnosed with ER-positive breast cancers, stage I–III between 1 July 2006 and 30 June 2009

Characteristics	Adherent (N = 3 668)		Non-adherent (N = 977)		OR (95% CI)		
	No.	%	No.	%	Unadjusted	Adjusted for age	Adjusted for age and education
Patient-related							
Age at diagnosis (years)							
< 50	685	18.7	186	19.0	1.13 (0.93–1.37)	–	–
50–64	1630	44.4	392	40.1	1.0 (Ref)		
≥ 65	1353	36.9	399	40.8	1.23 (1.05–1.43)		
Menopausal status							
Premenopausal	723	19.7	196	20.1	1.0 (Ref)	1.0 (Ref)	1.0 (Ref)
Postmenopausal	2610	71.2	714	73.1	1.01 (0.85–1.21)	1.27 (0.94–1.72)	1.28 (0.95–1.73)
Unknown	335	9.1	67	6.9	–	–	–
Baseline CCI							
0	3121	85.1	809	82.8	1.0 (Ref)	1.0 (Ref)	1.0 (Ref)
1	355	9.7	108	11.1	1.17 (0.93–1.47)	1.14 (0.90–1.43)	1.15 (0.90–1.44)
2	130	3.5	36	3.7	1.07 (0.72–1.54)	0.98 (0.66–1.43)	0.99 (0.67–1.44)
3+	62	1.7	24	2.5	1.49 (0.91–2.37)	1.37 (0.83–2.20)	1.40 (0.85–2.24)
FU CCI							
0	2650	72.2	647	66.2	1.0 (Ref)	1.0 (Ref)	1.0 (Ref)
1	553	15.1	180	18.4	1.33 (1.10–1.61)	1.30 (1.07–1.58)	1.31 (1.08–1.59)
2	277	7.6	86	8.8	1.27 (0.98–1.64)	1.21 (0.92–1.57)	1.22 (0.93–1.58)
3+	188	5.1	64	6.6	1.39 (1.03–1.87)	1.29 (0.94–1.74)	1.30 (0.95–1.76)
Increase in CCI							
0	3021	82.4	767	78.5	1.0 (Ref)	1.0 (Ref)	1.0 (Ref)
1	440	12.0	129	13.2	1.15 (0.93–1.42)	1.10 (0.88–1.37)	1.11 (0.89–1.37)
2+	207	5.6	81	8.3	1.54 (1.17–2.01)	1.43 (1.08–1.88)	1.43 (1.08–1.88)
Prediagnostic HRT							
No	3393	92.5	849	86.9	1.0 (Ref)	1.0 (Ref)	1.0 (Ref)
Yes	275	7.5	128	13.1	1.86 (1.48–2.32)	1.99 (1.58–2.49)	1.98 (1.58–2.48)
Baseline analgesics							
No	2748	74.9	702	71.9	1.0 (Ref)	1.0 (Ref)	1.0 (Ref)
Yes	920	25.1	275	28.1	1.17 (1.00–1.37)	1.16 (0.99–1.36)	1.17 (1.00–1.37)
Baseline hypnotics and sedatives							
No	3189	86.9	808	82.7	1.0 (Ref)	1.0 (Ref)	1.0 (Ref)
Yes	479	13.1	169	17.3	1.39 (1.15–1.68)	1.38 (1.14–1.67)	1.37 (1.13–1.66)
Baseline antidepressants							
No	3301	90.0	868	88.8	1.0 (Ref)	1.0 (Ref)	1.0 (Ref)
Yes	367	10.0	109	11.2	1.13 (0.90–1.41)	1.13 (0.90–1.42)	1.13 (0.90–1.42)
Baseline GI drugs							
No	2928	79.8	834	85.4	1.0 (Ref)	1.0 (Ref)	1.0 (Ref)
Yes	740	20.2	143	14.6	0.68 (0.56–0.82)	0.68 (0.55–0.83)	0.68 (0.55–0.83)
Baseline vaginal oestrogen							
No	3577	97.5	948	97.0	1.0 (Ref)	1.0 (Ref)	1.0 (Ref)
Yes	91	2.5	29	3.0	1.20 (0.77–1.81)	1.18 (0.75–1.78)	1.17 (0.75–1.78)
Socioeconomic factors							
Marital status							
Married	2008	54.7	437	44.7	1.0 (Ref)	1.0 (Ref)	1.0 (Ref)
Single/divorced/widowed	1656	45.1	539	55.2	1.50 (1.30–1.72)	1.42 (1.23–1.64)	1.42 (1.23–1.64)
Unknown	4	0.1	1	0.1	–	–	–
Education							
Low	900	24.5	225	23.0	1.0 (Ref)	1.0 (Ref)	–
Medium	1503	41.0	399	40.8	1.06 (0.88–1.28)	1.17 (0.97–1.42)	–
High	1241	33.8	345	35.3	1.11 (0.92–1.34)	1.25 (1.02–1.53)	–
Unknown	24	0.7	8	0.8	–	–	–
Health system related							
Region							
Stockholm–Gotland	1376	37.5	446	45.6	1.0 (Ref)	1.0 (Ref)	1.0 (Ref)
Uppsala-Örebro	1751	47.7	431	44.1	0.76 (0.65–0.88)	0.74 (0.64–0.86)	0.75 (0.64–0.87)
Northern Sweden	541	14.7	100	10.2	0.57 (0.45–0.72)	0.58 (0.45–0.73)	0.59 (0.46–0.74)
Hospital type for surgery							
University hospital	1004	27.4	244	25.0	1.0 (Ref)	1.0 (Ref)	1.0 (Ref)
Other hospitals	2655	72.4	732	74.9	1.13 (0.97–1.34)	1.12 (0.95–1.32)	1.12 (0.96–1.32)
Unknown	9	0.2	1	0.1	–	–	–
Hospital type for follow-up							
University hospital	1452	39.6	360	36.8	1.0 (Ref)	1.0 (Ref)	1.0 (Ref)
Other hospitals	2007	54.7	548	56.1	1.10 (0.95–1.28)	1.12 (0.96–1.30)	1.12 (0.97–1.30)
Unknown	209	5.7	69	7.1	–	–	-
Disease-related							
Tumour size (mm)							
< 20	2255	61.5	686	70.2	1.0 (Ref)	1.0 (Ref)	1.0 (Ref)
20–50	1208	32.9	261	26.7	0.71 (0.61–0.83)	0.65 (0.55–0.77)	0.65 (0.56–0.77)
≥ 50	126	3.4	18	1.8	0.47 (0.28–0.75)	0.43 (0.25–0.70)	0.44 (0.25–0.70)
Unknown	79	2.2	12	1.2	–	–	–
Lymph node metastasis							
No	3103	84.6	883	90.4	1.0 (Ref)	1.0 (Ref)	1.0 (Ref)
Yes	534	14.6	79	8.1	0.52 (0.40–0.66)	0.51 (0.39–0.65)	0.51 (0.40–0.65)
Unknown	31	0.8	15	1.5	–	–	–
Tumour grade							
I	827	22.5	289	29.6	1.0 (Ref)	1.0 (Ref)	1.0 (Ref)
II	2022	55.1	511	52.3	0.72 (0.61–0.85)	0.70 (0.59–0.83)	0.70 (0.59–0.83)
III	747	20.4	156	16.0	0.60 (0.48–0.74)	0.58 (0.47–0.73)	0.58 (0.47–0.73)
Unknown	72	2.0	21	2.1	–	–	–
PR status							
Pr−	659	18.0	167	17.1	1.0 (Ref)	1.0 (Ref)	1.0 (Ref)
Pr+	2988	81.5	806	82.5	1.06 (0.89–1.29)	1.05 (0.88–1.28)	1.06 (0.88–1.29)
Unknown	21	0.6	4	0.4	–	–	–
HER2 status							
Her2−	2772	75.6	725	74.2	1.0 (Ref)	1.0 (Ref)	1.0 (Ref)
Her2+	344	9.4	55	5.6	0.61 (0.45–0.82)	0.61 (0.45–0.81)	0.61 (0.45–0.82)
Unknown	552	15.0	197	20.2	–	–	-
Treatment-related							
Type of primary surgery							
Sector resection	2325	63.4	659	67.5	1.0 (Ref)	1.0 (Ref)	1.0 (Ref)
Mastectomy	1281	34.9	304	31.1	0.84 (0.72–0.97)	0.75 (0.64–0.87)	0.75 (0.64–0.87)
Other types	7	0.2	5	0.5	2.52 (0.74–7.92)	2.51 (0.74–7.92)	2.48 (0.73–7.84)
Not operated	49	1.3	6	0.6	0.43 (0.17–0.94)	0.43 (0.16–0.94)	0.43 (0.17–0.94)
Unknown	6	0.2	3	0.3	–	–	–
Radiotherapy							
No	1021	27.8	309	31.6	1.0 (Ref)	1.0 (Ref)	1.0 (Ref)
Yes	2647	72.2	668	68.4	0.83 (0.72–0.97)	0.95 (0.80–1.12)	0.94 (0.80–1.12)
Adjuvant chemotherapy							
No	2577	70.3	824	84.3	1.0 (Ref)	1.0 (Ref)	1.0 (Ref)
Yes	1091	29.7	153	15.7	0.44 (0.36–0.53)	0.42 (0.35–0.52)	0.43 (0.35–0.52)
Type of endocrine treatment							
Tamoxifen only	1805	49.2	536	54.9	1.0 (Ref)	1.0 (Ref)	1.0 (Ref)
AI only	640	17.4	138	14.1	0.73 (0.59–0.89)	0.72 (0.58–0.89)	0.72 (0.58–0.89)
Tamoxifen + AI	1223	33.3	303	31.0	0.83 (0.71–0.98)	0.85 (0.73–1.00)	0.86 (0.73–1.01)
First endocrine treatment							
Tamoxifen	757	61.9	221	72.9	1.0 (Ref)	1.0 (Ref)	1.0 (Ref)
AI	466	38.1	82	27.1	0.60 (0.45–0.79)	0.54 (0.41–0.72)	0.54 (0.41–0.72)
ET switch							
No	2445	66.7	674	69.0	1.0 (Ref)	1.0 (Ref)	1.0 (Ref)
Yes	1223	33.3	303	31.0	0.90 (0.77–1.05)	0.93 (0.79–1.08)	0.93 (0.80–1.09)
FU analgesics							
No	2040	55.6	450	46.1	1.0 (Ref)	1.0 (Ref)	1.0 (Ref)
Yes	1628	44.4	527	53.9	1.47 (1.27–1.69)	1.41 (1.22–1.63)	1.43 (1.24–1.66)
FU hypnotics and sedatives							
No	2712	73.9	634	64.9	1.0 (Ref)	1.0 (Ref)	1.0 (Ref)
Yes	956	26.1	343	35.1	1.53 (1.32–1.78)	1.50 (1.28–1.75)	1.49 (1.28–1.74)
FU antidepressants							
No	2852	77.8	713	73.0	1.0 (Ref)	1.0 (Ref)	1.0 (Ref)
Yes	816	22.2	264	27.0	1.29 (1.10–1.52)	1.27 (1.08–1.49)	1.27 (1.08–1.50)
FU GI drugs							
No	2932	79.9	726	74.3	1.0 (Ref)	1.0 (Ref)	1.0 (Ref)
Yes	736	20.1	251	25.7	1.38 (1.17–1.62)	1.30 (1.10–1.54)	1.31 (1.10–1.55)
FU vaginal oestrogen							
No	3261	88.9	836	85.6	1.0 (Ref)	1.0 (Ref)	1.0 (Ref)
Yes	407	11.1	141	14.4	1.35 (1.10–1.66)	1.34 (1.08–1.65)	1.33 (1.08–1.64)

*OR* odds ratio, *CI* confidence interval, *FU* follow-up, *AI* aromatase inhibitors, *GI* gastro-intestinal

### Patient-related factors

The likelihood of non-adherence increased with older age (OR 1.23, 95% CI 1.05–1.43 for age 65 and older compared to 50–64), an increase in the comorbidity burden during and at the end of 5-year observation (e.g. OR 1.54, 95% CI 1.17–2.01 for gain of 2 or more in CCI compared to 0 during follow-up), prior use of HRT (OR 1.86, 95% CI 1.48–2.32), use of analgesics (OR 1.17, 95% CI 1.00–1.37) or hypnotics and sedatives (OR 1.39, 95% CI 1.15–1.68) at baseline. Use of GI drugs at baseline was inversely associated with non-adherence (OR 0.68, 95% CI 0.56–0.82). Following adjustment for age, associations remained for increases in CCI during follow-up, prior HRT use, use of hypnotics and sedatives and GI drugs at baseline (Table 1). Further adjustment for education did not alter these results.

### Socioeconomic factors

There were differences in ET adherence across indicators of socioeconomic standing as reflected by greater odds of non-adherence by non-married/single status (unmarried versus married age-adjusted OR 1.42, 95% CI 1.23–1.64). Also, the likelihood of non-adherence was elevated in women with high compared to low educational level (age-adjusted OR 1.25, 95% CI 1.02–1.53), an association that remained virtually unchanged following adjustments for tumour size, presence of lymph node metastasis and pre-diagnostic use of HRT (data not shown).

### Health system factors

While there was evidence of regional variations with the lowest odds of non-adherence observed in Northern Sweden (age-adjusted OR 0.58, 95% CI 0.45–0.73), no differences were observed between university hospitals and other types of hospitals. Results remained unchanged following adjustment for education (Table 1).

### Disease-related factors

The presence of lymph node metastasis (OR 0.52, 95% CI 0.40–0.66), higher tumour grade (e.g. OR 0.60, 95% CI 0.48–0.74 for grade III compared to I) and HER2-positive tumours (OR 0.61, 95% CI 0.45–0.82) were associated with lower odds of non-adherence to ET. These associations remained following adjustment for age and education (Table 1).

### Treatment

We found lower odds of non-adherence in women undergoing mastectomy and in women without a record of surgery (*n* = 55) compared to sector resection (age-adjusted OR 0.75, 95% CI 0.64–0.87 and 0.43, 95% CI 0.16–0.94, respectively), use of AI or both AI and tamoxifen compared to tamoxifen only (age-adjusted OR 0.72, 95% CI 0.58–0.89 and 0.85, 95% CI 0.73–1.00, respectively), starting out ET with AI rather than tamoxifen (age-adjusted OR 0.54, 95% CI 0.41–0.72), and in women receiving adjuvant chemotherapy (age-adjusted OR 0.42 95% CI 0.35–0.52).

Compared to non-users, odds of non-adherence were elevated in women using hypnotics and sedatives (age- adjusted OR 1.50, 95% CI 1.28–1.75), antidepressants (age-adjusted OR 1.27, 95% CI 1.08–1.49), GI drugs (age-adjusted OR 1.30, 95% CI 1.10–1.54) and vaginal oestrogens (age- adjusted OR 1.34, 95% CI 1.08–1.65) during follow-up. These associations remained following adjustment for education (Table 1). Results were similar, albeit weaker when women without ET dispensation during the final year of follow-up were excluded (data not shown).

### Comorbidity burden

In a final step, we assessed whether non-adherence varied between women who did not have a specific co-existing medical condition, those who had the comorbidity prior to treatment, and women diagnosed with the condition during follow-up. These analyses were repeated for all disease entities included in the CCI domain, and results for the ten major comorbidities are shown in Table 2. The most common co-existing conditions observed included chronic pulmonary disease, cardiovascular disease and diabetes. Non-adherence was more common in women with a first record of chronic pulmonary disease during follow-up (44 out of 141 women were non-adherent = 31%) compared to those who never had it (21%) or were diagnosed with the condition before start of ET (22%), with significant differences demonstrated by Fisher’s exact test (*P* = 0.01). A higher proportion of non-adherent women (*P* = 0.007) was also observed in those diagnosed with myocardial infarction (MI) during follow-up (37%) compared to women without a history of MI (21%) or with a record of MI before ET (33%). No difference in the proportion of non-adherence was observed across women who had diabetes before diagnosis, during follow-up, or those without the condition.

**Table 2:** Non-adherence across prevalence of ten leading co-morbidities

	N non-adherent/N adherent			*P* value*
	Never	At baseline	During follow-up	
Chronic pulmonary disease	881/3377	55/191	44/97	0.01
Cerebrovascular disease	907/3459	40/108	33/98	0.09
Diabetes without complications	908/3405	49/161	23/99	0.62
Congestive heart failure	937/3535	18/56	25/74	0.43
Connective tissue and rheumatic disease	945/3555	19/72	16/38	0.30
Myocardial infarction	949/3607	14/29	17/29	0.007
Diabetes with complications	960/3606	12/36	8/23	0.59
Other cancer(s)	960/3583	20/82	0/0	0.81
Dementia	960/3611	4/10	16/44	0.40
Peripheral vascular disease	964/3606	10/24	6/35	0.32
Peptic ulcer disease	965/3614	2/23	13/28	0.07
Renal disease	967/3627	5/12	8/26	0.55
Paraplegia and hemiplegia	970/3642	4/10	6/13	0.33
Moderate or severe liver disease	978/3656	1/3	1/6	0.99

*Fisher’s exact *P* value

## Discussion

In Swedish women undergoing adjuvant endocrine treatment for ER-positive non-metastatic breast cancer, we found a broad range of factors associated with non-adherence to ET including age, region, increases in comorbidity burden, marital status, educational level, use of HRT and symptom-relieving drugs, tumour characteristics, adjuvant chemotherapy and type of ET.

While we found no strong association between younger age and non-adherence to ET, our results support earlier evidence of lower adherence in both younger and older age groups [6, 7]. It has been suggested that not only treatment side effects, but also fertility concerns contribute to low adherence and discontinuation in young (< 45 years) women [14]. In older age groups, multi-morbidity has been suggested to explain lower rates of adherence [15]. Few studies to date have addressed the possible role of specific comorbidities on adherence to ET. In the present study, we found evidence of higher odds of non-adherence in women among whom the comorbidity burden increased during follow-up, particularly chronic pulmonary disease and myocardial infarction. Interestingly, long-term use of cardiovascular drugs, i.e. antihypertensives and statins has been linked to greater adherence, [16] whereas non-adherence to chronic medications including cardiovascular drugs was associated with non-adherence to ET [17]. Taken together, these findings point to complex associations between specific age-related comorbidities, poly-pharmacy and adherence to ET.

We found that women with a previous history of HRT use were less likely to adhere to ET. Earlier findings indicate that HRT use is associated with an increased risk of adverse effects from ET [18, 19], possibly reflecting withdrawal effects from discontinuation of HRT that add to side effects from ET. An alternative explanation is that women with a previous history of HRT use, on a group level are more prone to develop postmenopausal symptoms as side effects from ET. Or it could be a combination of these two suggested mechanisms.

Chemotherapy preceding ET was associated with better adherence, a finding that may be explained by a more advanced tumour stage and a high motivation to remain adherent.

We also found associations between use of hypnotics and sedatives both at baseline and during follow-up, and non-adherence to ET, possibly reflecting that quality of life influences the likelihood to use ET as prescribed. However, further research is needed to confirm the direction of this association.

In part, the present results support previous evidence of a role of socioeconomic factors as predictors of non-adherence to ET [6]. Our finding of an association between being unmarried or living alone and poorer adherence may be mediated by low social support [20]. We found minor differences in adherence between educational groups; if anything, the likelihood of non-adherence was elevated in women with the highest compared to low education. In previous studies, low adherence in low-income populations have been reported [21, 22]. A US-based study on patterns of use of AIs within 1 year of start of treatment found that high out-of-pocket prescription costs negatively affected adherence in commercially insured women with breast cancer [23] In Sweden, there is a cap limitation on of out-of-pocket cost for medication, which may explain the lack of an association in our study between low education and non-adherence. Our finding of an elevated risk of non-adherence in the group of women with the highest education is difficult to explain, but may reflect informed patient decision-making regarding risk versus benefits of adjuvant endocrine therapy.

We found some evidence of regional differences in adherence proportions. Regional differences may reflect variation in patterns of breast cancer management,[24] including follow-up, repeat visits and counselling. Despite the existence of Swedish national guidelines for breast cancer management, reports from the National Breast Cancer Quality Register have documented regional differences in some dimensions of provision of breast cancer care [25]. In a recent register-based study conducted in three counties in Southern Sweden, all with systems of yearly follow-up of women prescribed ET, non-adherence was considerable lower than that observed in our study [12]. It is of importance to clarify which specific components of provider counselling and follow-up that improve adherence to ET.

We found that women that had undergone mastectomy, and those without a history of surgery, were less likely to be non-adherent compared to sector resected women. In women undergoing mastectomy, a more advanced tumour stage may explain an adherent medication-taking behaviour, in particular, since we also found evidence of better adherence in patients with large tumour size and grade, or lymph node metastasis. On the other hand, in women not undergoing surgery, ET may be the only treatment, for instance, elderly and/or frail women. Thus, a low rate of non-adherence in this group of women could reflect a high motivation to use ET in the absence of other treatments.

We did not have information on subjective patient-related factors which may influence the decision-making on ET [26]. Among these factors, concerns of adverse effects have been reported to be correlated with ET discontinuation [27]. Patient decision-making occurs not only prior to initiation of ET, but also during treatment [28]. This may indicate that information regarding ET benefits and implications needs to be communicated not only at start of treatment, but also at repeat visits or follow-up contacts. However, results from a recent systematic review indicate that behavioural interventions aimed to improve adherence through patient education and follow-up reminders were unable to demonstrate any benefit [29]. Taken together, these findings indicate that improvement of ET adherence requires comprehensive approaches involving determinants beyond patient-dependent factors.

Strengths of our study included the population-based setting covering three Swedish health care regions. Individual level information was available for a broad range of potential determinants spanning from patient-related to health system-related factors. In contrast to many prior studies based on short follow-up periods, we were able to assess adherence during a 5-year period for all women. Furthermore, the availability of information on comorbidity added to prior evidence by pointing to a possible role of specific medical conditions that increase the likelihood of non-adherence to ET.

Several limitations need mentioning. We measured adherence by drug dispensation/filings of prescriptions rather than by actual use, which may result in misclassification. However, direct measurements of drug use is costly, and bias has also been reported with other indirect measures such as patient self-report and pill count, especially in patient with chronic diseases [30]. Combination of indirect measures of adherence, such as pharmacy data used in this study, has been shown to correlate better with clinical outcomes compared to individual level measures, [31] but its feasibility is unclear especially in the context of cancer.

Also, we were unable to explore the possible influence of psychological factors. However, we assessed associations between use of hypnotics, sedatives and antidepressants and non-adherence. Additionally, using objective measures of factors affecting adherence have been recommended instead of patient-centred measures which tend to stigmatise women as solely responsible for being non-adherent [32]. Future research also needs to investigate the causality of the observed associations to identify points of intervention to improve adherence.

## Conclusion

We conclude that predictors of non-adherence to ET extend beyond patient-related factors, supporting the notion that aspects of medication taking behaviours need to be addressed in a multidimensional fashion. Subgroups of women more likely to be non-adherent may benefit from tailored information on treatment benefit and risks, monitoring and repeated counselling throughout the planned course of endocrine treatment. Prospective studies are needed to determine the benefit of specific components of interventions to improve adherence.
